# Optimal antisense target reducing *INS* intron 1 retention is adjacent to a parallel G quadruplex

**DOI:** 10.1093/nar/gku507

**Published:** 2014-06-17

**Authors:** Jana Kralovicova, Ana Lages, Alpa Patel, Ashish Dhir, Emanuele Buratti, Mark Searle, Igor Vorechovsky

**Affiliations:** 1 University of Southampton, Faculty of Medicine, Southampton SO16 6YD, UK; 2 University of Nottingham, School of Chemistry, Centre for Biomolecular Sciences, Nottingham NG7 2RD, UK; 3 ICGEB, Padriciano 99, 34149 Trieste, Italy

## Abstract

Splice-switching oligonucleotides (SSOs) have been widely used to inhibit exon usage but antisense strategies that promote removal of entire introns to increase splicing-mediated gene expression have not been developed. Here we show reduction of *INS* intron 1 retention by SSOs that bind transcripts derived from a human haplotype expressing low levels of proinsulin. This haplotype is tagged by a polypyrimidine tract variant *rs689* that decreases the efficiency of intron 1 splicing and increases the relative abundance of mRNAs with extended 5' untranslated region (5' UTR), which curtails translation. Co-expression of haplotype-specific reporter constructs with SSOs bound to splicing regulatory motifs and decoy splice sites in primary transcripts revealed a motif that significantly reduced intron 1-containing mRNAs. Using an antisense microwalk at a single nucleotide resolution, the optimal target was mapped to a splicing silencer containing two pseudoacceptor sites sandwiched between predicted RNA guanine (G) quadruplex structures. Circular dichroism spectroscopy and nuclear magnetic resonance of synthetic G-rich oligoribonucleotide tracts derived from this region showed formation of a stable parallel 2-quartet G-quadruplex on the 3' side of the antisense retention target and an equilibrium between quadruplexes and stable hairpin-loop structures bound by optimal SSOs. This region interacts with heterogeneous nuclear ribonucleoproteins F and H that may interfere with conformational transitions involving the antisense target. The SSO-assisted promotion of weak intron removal from the 5' UTR through competing noncanonical and canonical RNA structures may facilitate development of novel strategies to enhance gene expression.

## INTRODUCTION

Most eukaryotic genes contain intervening sequences or introns that must be accurately removed from primary transcripts to create functional mRNAs capable of encoding proteins (1). This process modifies mRNP composition in a highly dynamic manner, employing interdependent interactions of five small nuclear RNAs and a large number of proteins with conserved but degenerate sequences in the pre-mRNA (2). Intron splicing generally promotes mRNA accumulation and protein expression across species (3–5). This process can be altered by intronic mutations or variants that may also impair coupled gene expression pathways, including transcription, mRNA export and translation. This is best exemplified by introns in the 5' untranslated region (5' UTR) where natural variants or mutations modifying intron retention alter the relative abundance of transcripts with upstream open reading frames (uORFs) or other regulatory motifs and dramatically influence translation (6,7). However, successful sequence-specific strategies to normalize gene expression in such situations have not been developed.

Splice-switching oligonucleotides (SSOs) are antisense reagents that modulate intron splicing by binding splice-site recognition or regulatory sequences and competing with *cis-* and *trans*-acting factors for their targets (8). They have been shown to restore aberrant splicing, modify the relative expression of existing mRNAs or produce novel splice variants that are not normally expressed (8). Improved stability of targeted SSO-RNA duplexes by a number of SSO modifications, such as 2' -*O*-methyl and 2' -*O*-methoxyethyl ribose, facilitated studies exploring their therapeutic potential for a growing number of human disease genes, including *DMD* in muscular dystrophy (9,10), *SMN2* in spinal muscular atrophy (11), *ATM* in ataxia-telangiectasia (12) and *BTK* in X-linked agammaglobulinemia (13). Although such approaches are close to achieving their clinical potential for a restricted number of diseases (8), >300 Mendelian disorders resulting from mutation-induced aberrant splicing (14) and a growing number of complex traits may be amenable to SSO-mediated correction of gene expression.

Etiology of type 1 diabetes has a strong genetic component conferred by human leukocyte antigens (HLA) and a number of modifying non-HLA loci (15). The strongest modifier was identified in the proinsulin gene (*INS*) region on chromosome 11 (termed IDDM2) (15). Further mapping of this area suggested that *INS* is the most likely IDDM2 target (16), consistent with a critical role of this autoantigen in pathogenesis (17). Genetic risk to this disease at IDDM2 has been attributed to differential steady-state RNA levels from predisposing and protective *INS* haplotypes, potentially involving a minisatellite DNA sequence upstream of this gene (18,19). However, systematic examination of naturally occurring *INS* polymorphisms revealed haplotype-specific proinsulin expression levels in reporter constructs devoid of the minisatellite sequence, resulting from two variants in intron 1 (7), termed IVS1+5ins4 (also known as *rs3842740* or INS-69) and IVS1–6A/T (*rs689*, INS-27 or *Hph*I+/−) (16,20). The former variant activates a cryptic 5' splice site of intron 1 whereas adenine (A) at the latter variant, which resides 6 nucleotides upstream of the 3' splice site (3' ss), promotes intron retention, expanding the relative abundance of transcripts with extended 5' UTR (21). As compared to thymine (T), the A allele at IVS1–6A/T decreases affinity to pyrimidine-binding proteins *in vitro* and renders the 3' ss more dependent on the auxiliary factor of U2 small nuclear ribonucleoprotein (U2AF) (7), a heterodimer required for U2 binding, spliceosome assembly and 3' ss selection (22). Intron 1-containing transcripts are overrepresented in IVS1-6A-derived cDNA libraries prepared from insulin producing tissues (21), are exported from the nucleus (23) and contain a short, *Homininae*-specific uORF that co-evolved with relaxation of the 3' ss of intron 1 in higher primates (7). The lower proinsulin expression conferred by the A allele may lead to suboptimal presentation of proinsulin peptides in the foetal thymus and inadequate negative selection of autoreactive T cells, culminating in autoimmune destruction of insulin-producing β cells in the pancreas (7). However, no attempts have been made to correct the low efficiency of *INS* intron 1 removal from the IVS1-6A-containing pre-mRNAs and reduce intron retention to the levels observed for the disease-protective T allele.

In this study, we set out to search for SSOs that increase the efficiency of *INS* intron 1 splicing and repress splicing silencers or decoy splice sites in the pre-mRNA to enhance proinsulin expression. We report identification of SSOs reducing the relative abundance of intron 1-retaining transcripts, delineation of the optimized antisense target at a single-nucleotide resolution, evidence for formation of a parallel G-quadruplex adjacent to the antisense target sequence and identification of proteins that bind to this region.

## MATERIALS AND METHODS

### Antisense oligonucleotides

**Figure 1.**
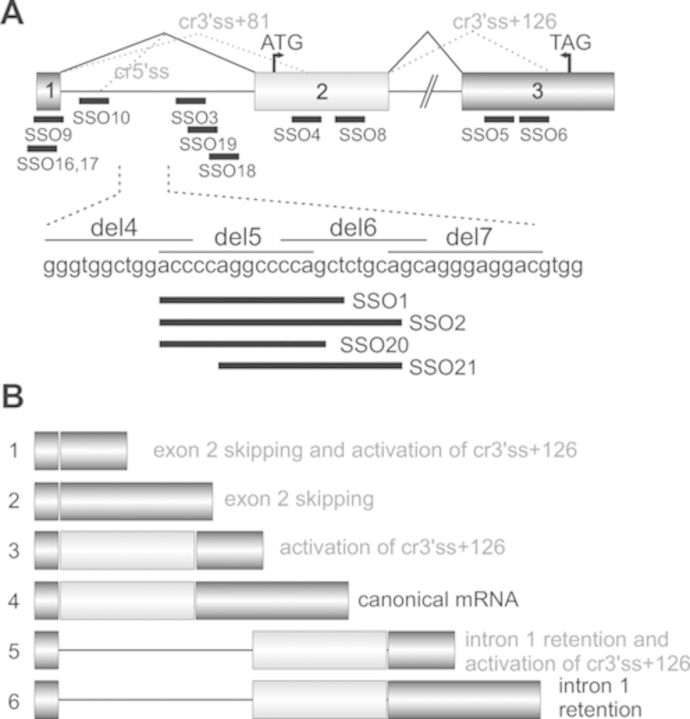
Location of SSOs in the human proinsulin gene. (**A**) Schematics of the *INS* reporter and its mRNA products. SSOs are shown as black horizontal bars below exons (numbered boxes) and below intron 1 (line); their sequences are in Supplementary Table S1. Start and stop codons are denoted by arrowheads. Canonical (solid lines) and cryptic (dotted lines) splicing is shown above the primary transcript; designation of cryptic splice sites is in grey. SSOs targeting intron 1 segments del4-del7 are shown in the lower panel. (**B**) mRNA isoforms (numbered 1–6) generated by the *INS* reporter construct. Description of isoforms that do not produce proinsulin is in grey.

SSOs were purchased from the MWG Biotech (Germany). All SSOs and scrambled controls had a full-length phosphorothioate backbone with 2' -*O*-methyl ribonucleotides at the second ribose position. Apart from *INS* SSOs and their scrambled versions, we employed SSOs that target other human genes as additional controls, as described (13). Location of each SSO is shown in Figure 1A and their sequences in Supplementary Table S1.

### Splicing reporter constructs

The wild-type splicing reporter carrying the type 1 diabetes-associated haplotype termed IC was reported previously (7,21). Each construct contains all *INS* exons and unabridged introns but differ in the length of the last exon. The IC reporters were cloned using primers D-C, D-F and D-B; IC D-B lacks the cryptic 3' ss of intron 2. The relative abundance of isoforms spliced to this site is lower for IC D-F than for IC D-C (7,21). To test SSOs targeting the cryptic 5' splice site of intron 1, the IC construct was modified by a 4-nt insertion at *rs3842740* to create a reporter termed IC-IVS1+5ins4. *TSC2* and *F9* constructs were reported previously (24). Plasmids were propagated in the *E. coli* strain DH5α and plasmid DNA was extracted using the Wizard Plus SV Miniprep kit (Promega, USA). Their inserts were completely sequenced to confirm the identity of each of the 14 intragenic natural variants and to exclude undesired mutations.

### Cell cultures and transfections

Human embryonic kidney 293 (HEK293), human hepatocellular liver carcinoma HepG2 and African green monkey COS7 cells were cultured in Dulbecco's modified Eagle medium, 10% fetal calf serum and penicillin/streptomycin (Life technologies, USA). Transient transfections were carried out as described (13), using jetPRIME (Polyplus, USA) according to manufacturer's recommendations. Downregulation of U2AF35 by RNA interference (RNAi) to induce cryptic 3' ss of intron 1 was performed with two hits of small interfering RNA (siRNA) U2AF35ab, as reported earlier (7,25); siRNA duplex targeting DHX36 was as described (26). The second hit was applied 24 h before the addition of SSOs and/or reporter. Cell cultures were harvested 24 h after addition of reporter constructs.

### Analysis of spliced products

Total RNA was extracted with TRI Reagent and treated with DNase (Life technologies, USA). The first-strand cDNA was reverse transcribed using oligo-(dT)_15_ primers and Moloney murine virus reverse transcriptase (Promega, USA). Polymerase chain reaction (PCR) was carried out with a combination of a vector-specific primer PL3 and primer E targeting the 3' UTR, as reported previously (7). PCR products were separated on polyacrylamide gels and their signal intensity was measured as described (27). The identity of each mRNA isoform was confirmed by Sanger nucleotide sequencing.

### Circular dichroism and nuclear magnetic resonance spectroscopy

Oligoribonucleotides for circular dichroism (CD) and nuclear magnetic resonance (NMR) were purchased from Thermo Scientific, deprotected according to manufacturer's instructions, lyophilized and stored at −20°C. Stock solutions were prepared from the desalted, lyophilized samples by resuspending in milliQ water or KCl buffer (100 mM KCl, 10 mM K_2_HPO_4_/KH_2_PO_4_, pH 7.0, milliQ water) to a final concentration of 2–4 μM.

CD spectra were acquired using a PiStar-180 spectrophotometer (Applied Photophysics Ltd, Surrey, UK), equipped with a LTD6G circulating water bath (Grant Instruments, UK) and thermoelectric temperature controller (Melcor, USA). Samples were heated in the cell to 95ºC for a total period of 15 min, samples were then annealed by allowing to cool to room temperature for a minimum period of 4 h. CD spectra were recorded over a wavelength range of 215–340 nm using a 1 cm path length strain-free quartz cuvette and at the temperatures indicated. Data points recorded at 1 nm intervals. A bandwidth of 3 nm was used and 5000 counts acquired at each point with adaptive sampling enabled. Each trace is shown as the mean of three scans (±SD). CD temperature ramps were acquired at 265 nm corresponding to the band maxima of the folded quadruplex species. Ranges between 5 and 99ºC were used, with points acquired at 0.5ºC intervals with a 120–180 s time-step between 0.5ºC increments. Points were acquired with 10 000 counts and adaptive sampling enabled. Heating and cooling studies were compared to check for hysteresis and overall reversibility.

NMR spectra (^1^H) were collected at 800 MHz using a Bruker Avance III spectrometer with a triple resonance cryoprobe. Standard Bruker acquisition parameters were used. Data were collected using Topspin (v. 3.0) and processed in CCPN Analysis (v. 2.1).

### Pull-down assays and western blotting

*In vitro* transcription was carried out using MEGAshortscript™ T7 (LifeTechnologies, USA) and T7-tagged PCR products amplified with primers 5' -ATTAATACGACTCACTATAGGGCTCAGGGTTCCAGG and 5' -TGCAGCAGGGAGGACG, and DNA of the indicated plasmids as a template. Indicated synthetic RNAs were purchased from Eurofins UK. Five hundred pmols of each RNA was treated with 5 mM sodium *m*-periodate and bound to adipic acid dihydrazide agarose beads (Sigma, USA). Beads with bound RNAs were washed three times in 2 ml of 2 M NaCl and three times in buffer D (20 mM HEPES–KOH, pH 7, 6.5% v/v glycerol, 100 m M KCl, 0.2 mM EDTA, 0.5 mM dithiothreitol), incubated with HeLa nuclear extracts and buffer D with heparin at a final concentration of 0.5 mg/ml. Unbound proteins were washed five times with buffer D. Bound proteins were separated on 10% sodium dodecyl sulphate-polyacrylamide gel electrophoresis, stained by Coomassie blue and/or blotted on to nitrocellulose membranes.

Western blotting was carried out as described (7). Antibodies were purchased from Sigma (hnRNP E1/E2, product number R4155, U2AF65, product number U4758 and SFRS2, product number S2320), Abcam (DHX36, product number ab70269) and Millipore (SC35, clone 1SC-4F11). Antiserum against hnRNP F and hnRNP H was a generous gift of Prof. Douglas Black, UCLA.

### Mass spectrometry analysis

Following trypsin digestion, samples were freeze dried and resuspended with 25 ul of 5% ACN/0.1% formic acid for mass spectrometry (MS). Peptides were analysed by LC/MS/MS using a Surveyor LC system and LCQ Deca XP Plus (ThermoScientific). The raw data files were converted into mascot generic files using the MassMatrix File Conversion Tool (Version 2.0; http://www.massmatrix.net) for input into the Mascot searching algorithm (Matrix Science).

### Enzymatic structural probing

RNA secondary structure determination with the use of limited V1 RNAse (Ambion), T1 RNAse (Ambion) and S1 nuclease (Fermentas) digestion has been described in detail elsewhere (28). Briefly, 1 μg aliquots of RNAs from the insertion (ins) and deletion (del) pre-mRNAs were digested with 0.002 U of RNAse V1, 0.05 U of RNAse T1 and 19 U of S1 nuclease in a 100 μl at 30°C for 10 min. An enzyme-free aliquot was used as a control (C). The cleaved RNAs were retrotranscribed according to standard protocols using antisense primers labeled with [^32^P]-ATP at the 5' end.

## RESULTS

### Antisense oligonucleotides that promote pre-mRNA splicing of a weak intron in 5' UTR

To identify SSOs capable of reducing retention of *INS* intron 1 and increase splicing-mediated translational enhancement, we designed a series of 2' -*O*-methyl-modifed phosphorothioate SSOs, individually co-expressed each SSO with a splicing reporter construct carrying haplotype IC in HEK293 cells and examined the relative abundance of exogenous mRNA products (Figure 1A and B). The IC haplotype in the reporter was devoid of the minisatellite sequence and contained a total of 14 polymorphic sites (7,20), including the A allele at *rs689*. This allele inhibits intron 1 splicing and yields lower proinsulin levels as compared to the more common T allele (21). SSOs targeting intron 1 and exon 2 were chosen in regions that showed the most prominent alterations of exon inclusion or intron retention in previous systematic deletion analyses of these sequences (7). SSOs in exon 3 were located between authentic 3' ss of intron 2 and a strong competing cryptic 3' ss 126 nt downstream to identify pre-mRNA motifs that modify their usage (Figure 1A).

**Figure 2.**
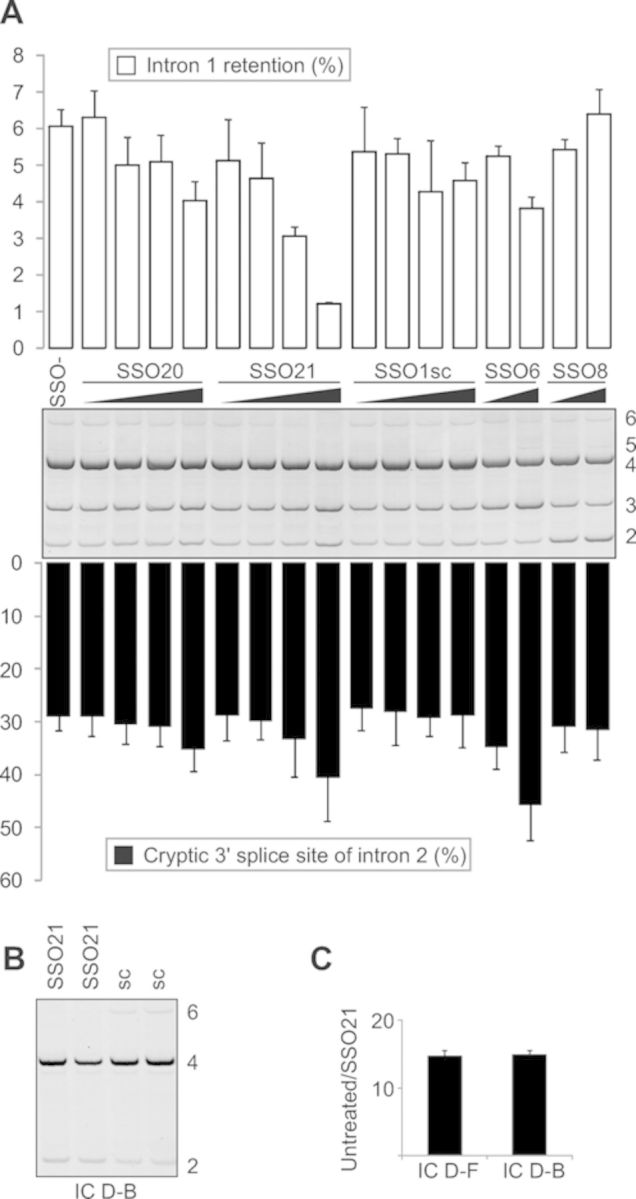
SSO-induced inhibition of *INS* intron 1 retention. (**A**) Cotransfection of the *INS* reporter construct (IC D-F) with the indicated SSOs into HEK293 cells. Spliced products described in Figure 1B are shown to the right. Bars represent percentage of intron 1-containing isoforms relative to natural transcripts (upper panel) or percentage of splicing to the cryptic 3' splice site of intron 2 relative to the total (lower panel). Error bars denote SD; sc, scrambled control; SSO-, ‘no SSO’ control. Final concentration of SSOs was 1, 3, 10 and 30 nM, except for SSO6 and SSO8 (10 and 30 nM). (**B**) SSO21-mediated promotion of intron 1 splicing in clones lacking the cryptic 3' ss of intron 2. RNA products are to the right. (**C**) A fold change in SSO21-induced intron 1 retention in transcripts containing and lacking the cryptic 3' ss of intron 2. The final concentration of SSO21 was 30 nM in duplicate transfection. Designation of the reporter constructs is at the bottom.

Of the initial set of 15 *INS* SSOs tested in HEK293 cells, 11 showed reproducible alterations in the relative abundance of mRNA isoforms (Supplementary Table S1). Intron 1 retention was significantly reduced by a single oligoribonucleotide SSO21 (*P* < 0.01, Mann-Whitney rank sum test; Figure 2A). SSO21 targeted intron 1 positions 59–74, encompassing a motif (termed del5) previously found to confer the largest reduction of intron retention upon deletion (7). The decrease in intron retention levels induced by SSO21 was dose-dependent (Figure 2A) and was also observed in HepG2 cells (Supplementary Figure S1) and *Chlorocebus aethiops* COS7 cells (data not shown), consistent with ubiquitous expression and a high degree of evolutionary conservation of spliceosome components that employ auxiliary splicing sequences (1,2).

**Figure 3.**
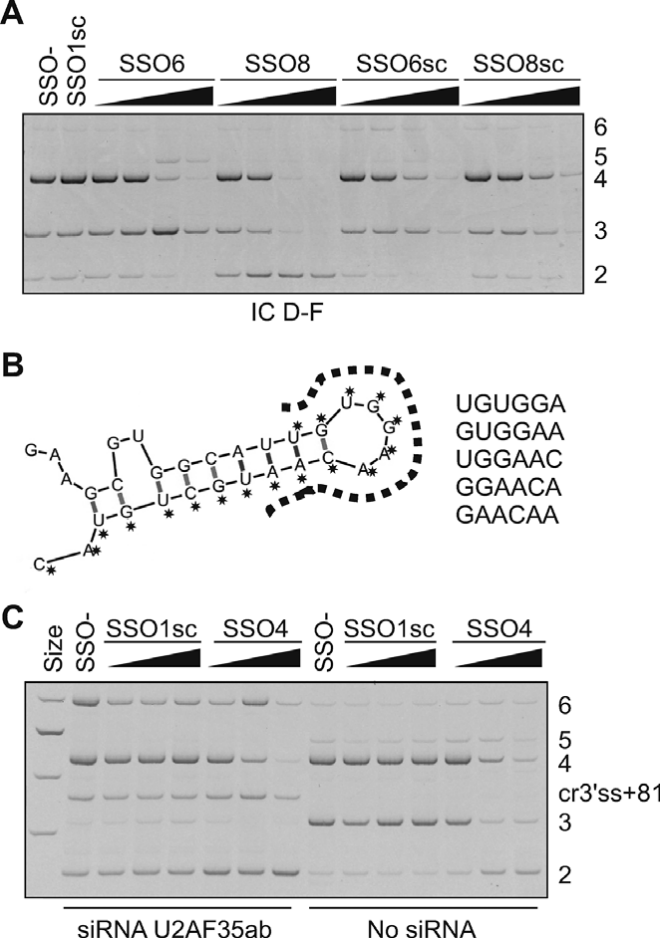
*INS* SSOs targeting cryptic 3' splice sites. (**A**) Activation of cryptic 3' ss of intron 2 (cr3' ss+126; Figure 1A) by SSO6 and promotion of exon 2 skipping by SSO8. Concentration of each SSO was 2, 10, 50 and 250 nM. SSOs are shown at the top, spliced products to the right, reporter at the bottom. (**B**) A predicted stable hairpin between the authentic and cryptic 3' ss of *INS* intron 2. Bases targeted by SSO6 are denoted by asterisks and predicted splicing enhancer hexamers (listed to the right) are denoted by a dotted line. (**C**) SSO4 does not prevent activation of cryptic 3' ss 81 base pairs downstream of its authentic counterpart (cr3' ss+81) in cells depleted of U2AF35 but induces exon skipping. The final concentration of each SSO in COS7 cells was 5, 20 and 80 nM. The final concentration of the siRNA duplex U2AF35ab (29) was 70 nM. The reporter was the same as in panel A.

In addition to reducing intron 1 retention, SSO21 promoted cryptic 3' ss of intron 2 (Figure 2A). However, this effect was also seen for other *INS* SSOs and for scrambled controls (Figure 3 and Supplementary Table S1), suggesting non-specific interactions. To confirm that the SSO21-induced enhancement of intron 1 splicing is not facilitated by the cryptic 3' ss of intron 2, we cotransfected this SSO with a shorter reporter lacking this site and retaining only the first 89 nucleotides of exon 3. Figure 2B shows that SSO21 was capable of promoting intron 1 splicing to the same extent as the reporter with longer exon 3. In contrast, the SSO21-induced decrease of intron retention was not observed for the reporter lacking the del5 segment (data not shown).

Apart from intron retention, we observed an increase of exon 2 skipping for five SSOs, including SSO8 that bound downstream of the cryptic 3' ss of intron 1 (cr3' ss+81; Figures 1 and 3C, Supplementary Table S1). This cryptic 3' ss was induced by RNAi-mediated depletion of the small subunit of U2AF (U2AF35) and was not reversed by a bridging oligoribonucleotide (SSO4) in cells lacking U2AF35; instead we observed exon 2 skipping (Figure 3C). Depletion of U2AF35 also repressed the cryptic 3' ss of intron 2. Taken together, we identified a single SSO that reduced *INS* intron 1 retention in several primate cell lines, consistent with a high degree of evolutionary conservation of spliceosome components that recognize auxiliary splicing sequences.

### Optimization of the intron retention target at the single-nucleotide level

**Figure 4.**
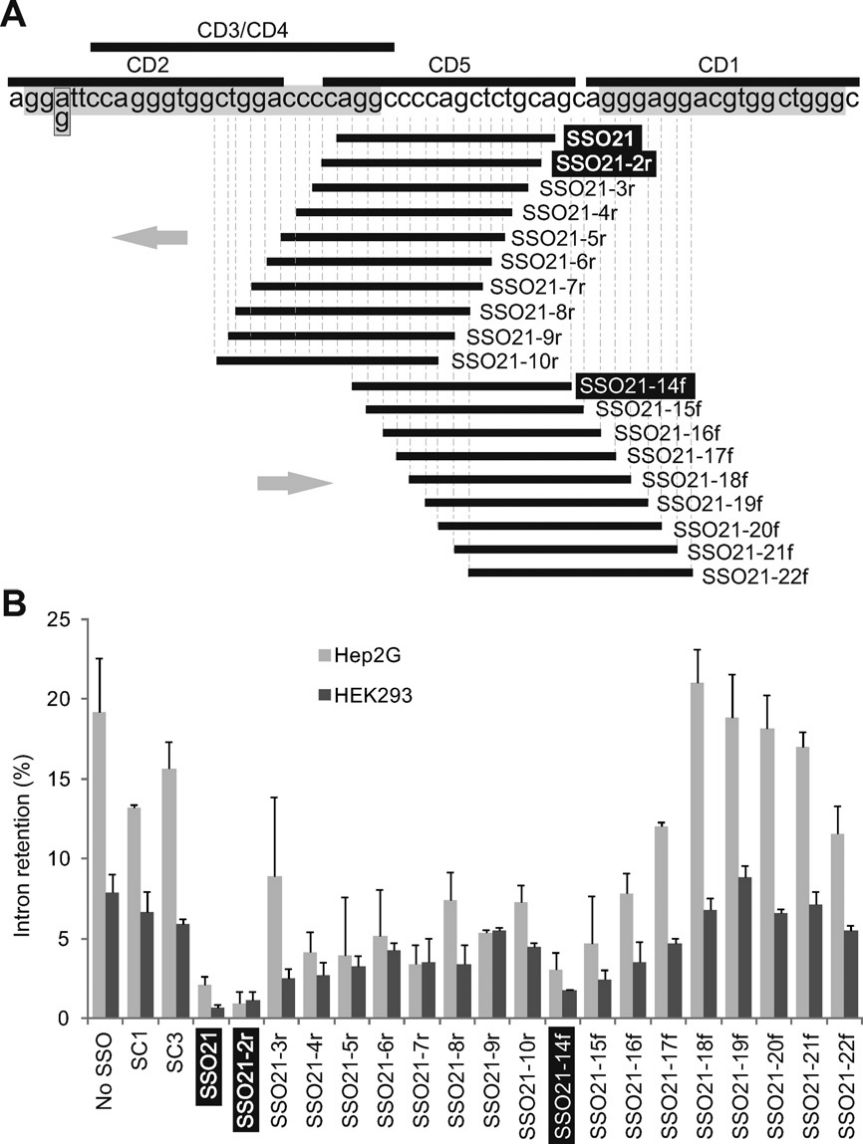
Optimization of the intron retention target by antisense microwalk at a single-nucleotide resolution. (**A**) Location of oligoribonucletoides. Microwalk SSOs and oligos used for CD/NMR are represented by horizontal black bars below and above the primary transcript, respectively. Intron 1 sequences predicted to form RNA G-quadruplexes are highlighted in grey. Microwalk direction is shown by grey arrows; winner oligos are highlighted in black. A box denotes a single nucleotide polymorphism reported previously (20). (**B**) Intron retention levels of each microwalk SSO in two cell lines. Error bars denote SDs obtained from two independent cotransfections with reporter IC D-F.

Interestingly, other SSOs designed to target the del5 segment did not reduce intron 1 retention, except for a small effect of SSO20 (Figures 1A and 2A). To test the importance of nucleotides flanking SSO21 and to map the optimal target at a single-base resolution, we carried out a detailed antisense microwalk in this region. We individually co-transfected the *INS* reporter with additional eighteen 16-mers bound 1–9 nucleotides 5' and 3' of SSO21 into HEK293 cells and examined their RNA products. Intron 1 retention was most repressed by SSO21 and by SSOs that were shifted by 1–2 nucleotides in each direction (Figure 4). In agreement with the initial screen, SSOs targeting more than one cytosine in the upstream run of four Cs (C4, see SSO1 and SSO2, Figure 1A) were not effective (SSO21–3r through SSO21–10r, Figure 4). In the opposite direction, SSOs targeting consecutive Gs, which are often found in intronic splicing enhancers (30–32), increased intron retention. Thus, the optimal antisense target for reducing retention of *INS* intron 1 was mapped at a single nucleotide resolution to a region previously identified as the most repressive by a systematic deletion analysis of the entire intron (7).

### Antisense target for intron retention is adjacent to a parallel RNA quadruplex

**Figure 5.**
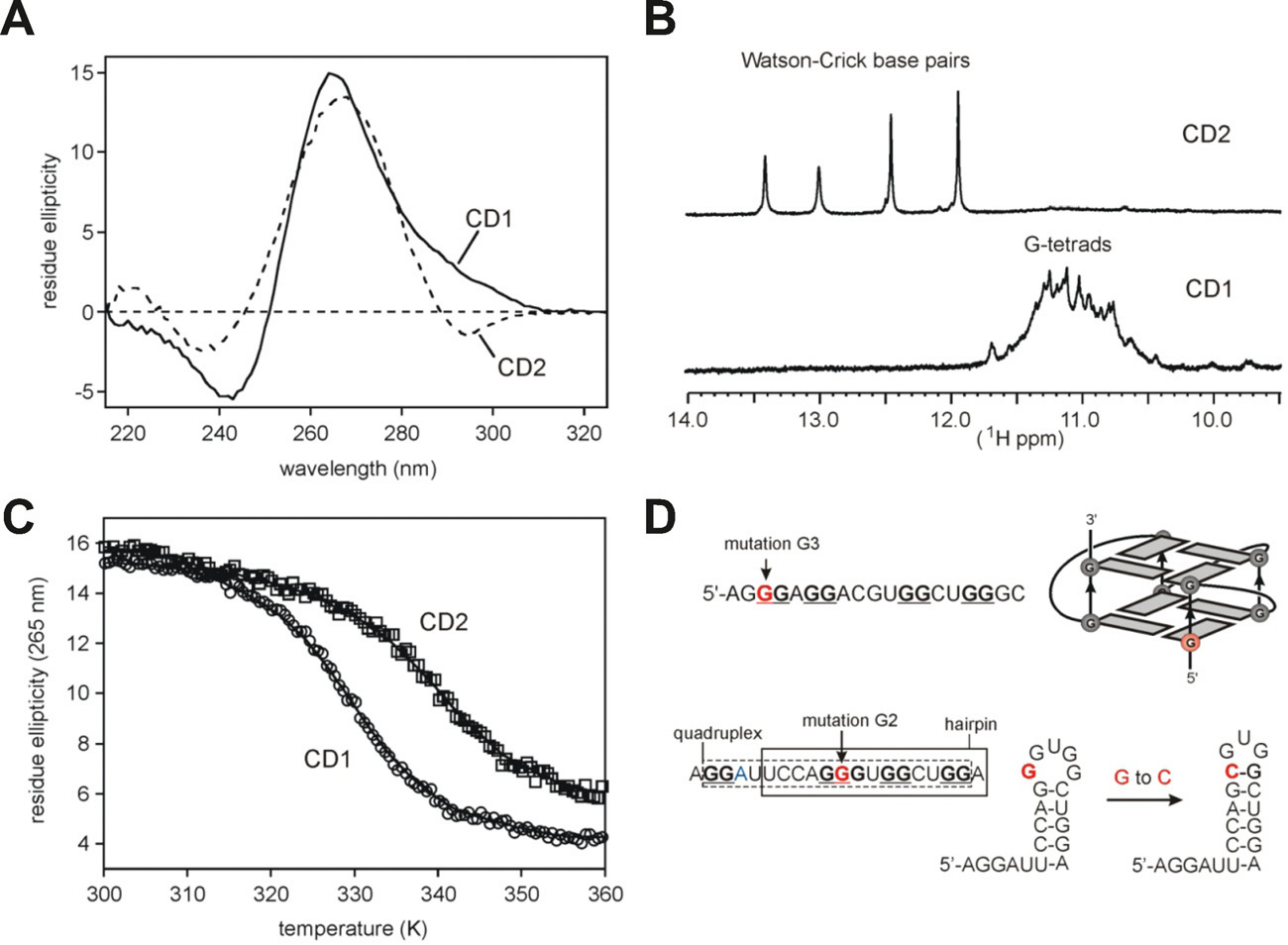
Biophysical characterization of RNA secondary structure formation. (**A**) Far-UV CD spectrum at 25°C for CD1 (19-mer) and CD2 (20-mer) RNAs, revealing ellipticity maximum at 265 and 270 nm, respectively. (**B**) ^1^H NMR spectra of CD1 and CD2 recorded at 800 MHz and 298 K showing characteristic groups of resonances from H-bonded G bases. (**C**) Sigmoidal CD melting curves for the two RNAs showing a transition mid-point at 56.8 ± 0.2°C and 69.0 ± 0.45°C, respectively. The two curves have been displaced slightly from each other for clarity. (**D**) The proposed parallel quadruplex structure with two stacked G-tetrads connected by short loop sequences for CD1 (top panel). Predicted hairpin structures for CD2 are shown at the bottom panel. G→C mutations are in red.

We noticed that the target was sandwiched between two intronic segments predicted to form stable RNA guanine (G) quadruplexes (intron 1 nucleotides 36–61 and 78–93; highlighted in Figure 4A). These structures are produced by stacking G-quartets that consist of four Gs organized in a cyclic Hoogsteen hydrogen bonding arrangement (33) and have been implicated in important cellular processes, including replication, recombination, transcription, translation (34,35) and RNA processing (36–40). To test if they are formed *in vitro*, we employed synthetic ribonucleotides derived from this region in CD spectroscopy that has been used widely to characterize DNA and RNA quadruplex structures (41–44). The CD spectrum of a downstream 19-mer (termed CD1) recorded between 215 and 330 nm at 25°C revealed strong positive ellipticity at 265 nm with negative intensity at around 240 nm, indicative of a parallel quadruplex (Figure 5A). To confirm the presence of a quadruplex, rather than other stable secondary structure motifs, we recorded UV absorbance spectra at 5°C and 95°C. The UV absorbance difference spectrum at the two temperatures (below and above the melting transition point) showed the characteristic hyperchromic shift at ∼295 nm (data not shown) and a double maximum at 240 nm and 280 nm, providing evidence for formation of a stable parallel-stranded RNA quadruplex *in vitro.* This was confirmed by ^1^H NMR studies of CD1 (Figure 5B) which showed a characteristic envelope of signals between 10 and 12 ppm corresponding to Hoogsteen H-bonded Gs within G-tetrad structures. Thermal stability measurements by CD produced a highly reversible sigmoidal co-operative unfolding transition with a *T_m_* = 56.8 ± 0.2°C (Figure 5C). Figure 5D (upper panel) shows a possible arrangement of the 19-mer into two stacked G-tetrads connected by relatively short loop sequences of 1–4 nucleotides.

### Conformational transition model for splicing inhibitory sequences in *INS* intron 1

CD of a synthetic 20-mer derived from a region upstream of the antisense target (termed CD2) also showed evidence of stable structure formation, giving a broader absorption envelope centered around 270 nm and a sigmoidal thermal unfolding transition (*T_m_* = 69.0 ± 0.45°C; Figure 5A). Unlike the downstream oligo CD1, no hyperchromic shift in the UV was found in the thermal difference spectrum (data not shown). However, a well-defined set of sharp signals in the ^1^H NMR spectrum between 12 and 14 ppm that differed from those for CD1 showed the formation of Watson–Crick H-bonded base pairs characteristic of double-stranded RNA (Figure 5B). Secondary structure predictions of overlapping intronic segments using Mfold suggested that the pre-mRNA forms stable local stem-loops; one of them was further stabilized by a G→C mutation (termed G2; Figure 5D, lower panel) that increased intron 1 retention (7). Another G→C substitution (termed G3) located further downstream and destabilizing the quadruplex structure (Figure 5D, upper panel) also repressed intron splicing (7). Finally, CD2 oligonucleotides containing either A or G at a single-nucleotide polymorphism (Figure 4A and (20) exhibited very similar CD spectra with well-defined melting transitions and *T_m_* values (data not shown), suggesting that the G and A alleles form the same structure.

**Figure 6.**
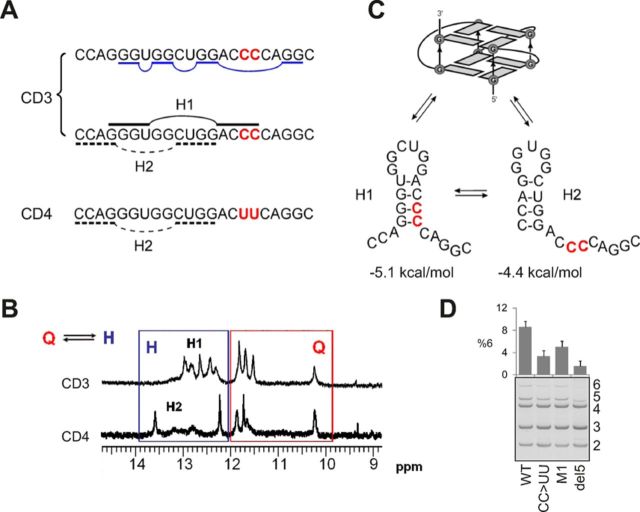
Conformational quadruplex/hairpin transitions involving the antisense target. (**A**) Schematic equilibrium between hairpin (black) and quadruplex (dark blue) structures proposed to form within the G-rich motif encompassing oligoribonucleotide CD3. CD4 contains a CC→UU mutation (in red). (**B**) The NMR spectrum in the 9–15 ppm region reveals imino proton signals corresponding to hydrogen bonded bases. The signals between 10 and 12 ppm are characteristic of Hoogsteen hydrogen bonded Gs within a G-tetrad (red box), while signals > 12 ppm are indicative of Watson–Crick A-U and G-C base pairs within hairpin structures (black box). In CD3, hairpin H1 is significantly populated, but mutations in CD4 destabilize H1 making H2 the major species, with both in equilibrium with the quadruplex structure. (**C**) Mfold predictions of two possible hairpins, consistent with the NMR data. (**D**) Reduction of intron retention upon destabilization of the hairpin structure by the CC→UU mutation. Error bars denote SD of a duplicate experiment with reporter IC D-C. Del5, the IC D-C reporter lacking segment del5 (Figure 1A); M1, a reporter containing two substitutions (Supplementary Table S2) to destabilize both the G-quadruplex and the stem-loop.

To test further the importance of a tentative equilibrium between canonical and noncanonical structures in intron splicing, we used a combination of CD, NMR and mutagenesis experiments (Figure 6). We synthesized an oligoribonucleotide CD3 encompassing the 5' end of the intron retention target and predicted stem-loops/quadruplex (Figures 4A and 6A). We also synthesised a mutated version CD4, which carried two C→U transitions destabilizing the hairpin but maintaining stability of the quadruplex. The same mutation was also introduced in our IC reporter construct transfected into HEK293 cells.

The NMR spectrum of CD3 revealed the co-existence of signals for both G-tetrad and canonical base-paired hairpin structures (termed H1 and H2) in equilibrium (Figure 6B and C ). We investigated the effects of Mg^2+^ on the conformational equilibrium between quadruplex and hairpin by adding 2 mM and then 6 mM MgCl_2_ to the buffered solution containing 100 mM KCl. As reported by Bugaut *et al.* (45), the conformational equilibrium was not significantly perturbed by the addition of Mg^2+^ in the presence of KCl. Thus, we observed formation of the RNA hairpin and quadruplex structures in an environment that mimics the cellular context where both K^+^ and Mg^2+^ ions were present at high concentrations. The CD melting curve showed a broad transition (*T_m_* = 79.9°C), consistent with multiple conformational states with different stabilities. The CC→UU mutation in CD4 resulted in the loss of NMR signals for H1 (Figure 6B) and a reduction in the *T_m_* by 13ºC, consistent with the selective destabilization of the more stable hairpin H1, leading to an increase in the population of H2 in equilibrium with the quadruplex. Transient transfections showed that the CC→UU mutation improved intron 1 splicing while a mutation termed M1 predicted to destabilize both the quadruplex and the hairpin had only a small effect (Figure 6D, Supplementary Table S2).

**Figure 7.**
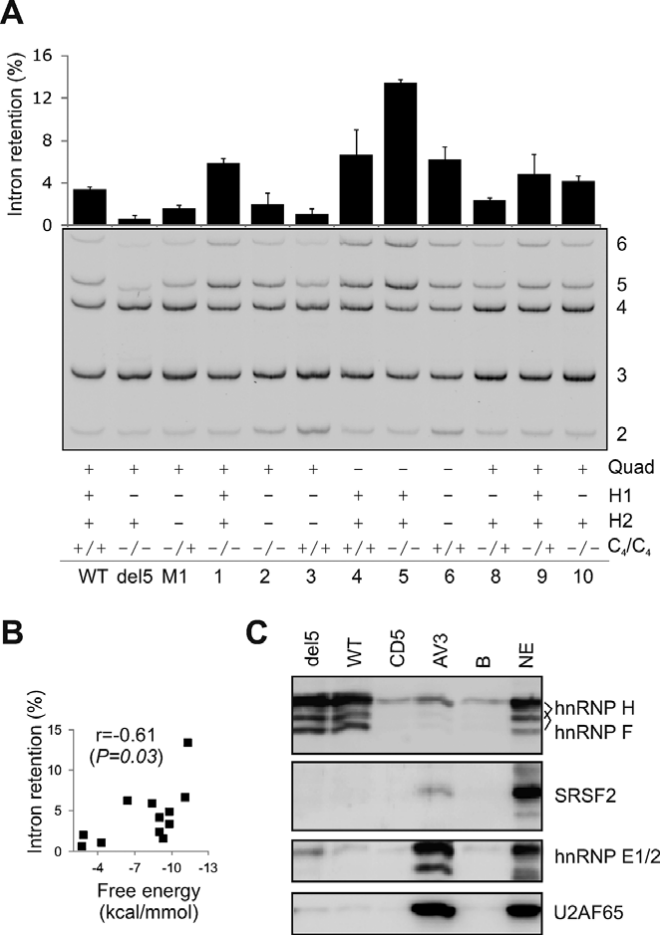
Identification of proteins that interact with pre-mRNAs encompassing the antisense target for intron retention. (**A**) Intron retention levels for wild type and mutated reporter constructs (IC D-C) following transient transfections into HEK293T cells. Mutations are shown in Supplementary Table S2. RNA products are to the right. The presence of predicted RNA quadruplexes, hairpins H1/H2 and the upstream and downstream C_4_ run are indicated below the gel figure. Error bars denote SDs obtained from two replicate experiments. (**B**) Intron retention levels of tested RNAs correlate with their predicted stabilities across the antisense target. (**C**) Western blot analysis of a pull-down assay with antibodies indicated to the right. NE, nuclear extracts; B, beads-only control; AV3, control RNA oligo containing a cytosine run and a 3' ss AG (7). The sequence of CD5 RNA is shown in Figure 4A.

To explore how the equilibrium of these structures affects intron splicing more systematically, we prepared a series of mutated constructs to destabilize/maintain predicted quadruplex, H1/H2 structures and two cytosine runs (Supplementary Table S2). Their transcripts showed significant differences in intron retention levels (Figure 7; *P* = 0.0001, Kruskal–Wallis one-way ANOVA on ranks). First, elimination of the G-quadruplex increased intron 1 retention, which was further enhanced by removing each cytosine run (cf. mutations 4–6 with the wild-type, *P* = 0.0004). These mutations appeared to have additive effects on intron retention (cf. wild-type versus mutations 1 or 9; 3 versus 2 and 4 versus 5). Second, the increased intron retention in the absence of the G-quadruplex was not altered by removing H1 and H2, but their elimination enhanced exon skipping (cf. isoform 2 for mutations 4 versus 6). Third, when only one of the two C4 runs was present, removal of H1 somewhat improved intron 1 splicing (cf. 8 versus 9), consistent with a statistically significant correlation between intron retention and predicted stability of tested RNAs (Figure 7B). The efficiency of intron splicing was thus controlled by conformational transitions between canonical and noncanonical structures in equilibrium.

### Protein–RNA interactions in the region targeted by winner SSOs

To identify proteins that interact with RNAs encompassing the antisense target and/or associated canonical and noncanonical structures, we carried out pull-down assays using wild type and del5 RNAs transcribed from T7-tagged PCR products, a synthetic RNA (CD5) representing the target sequence, and a control oligo containing a 3' ss CAG, termed AV3. Western blotting showed that both wild type and del5 transcripts bound hnRNPs F/H but this binding was absent for CD5 (Figure 7C). These proteins were also detected by MS/MS analysis of differentially stained fragments from pull down gels with wild type and del5 RNAs as compared to beads-only controls (data not shown). Two antibodies against SRSF2, which showed the highest score for putative binding activity among several SR proteins (Supplementary Figure S2), failed to detect any specific interaction (Figure 7C). Although the signal from hnRNP E1/E2, which constitute a major poly(C) binding activity in mammalian cells (46), was above background for del5 (Figure 7C), we observed no change in intron retention in cells lacking hnRNP E1/E2 (data not shown).

### Splicing pattern of G-rich and G-poor reporters upon DHX36 depletion

**Figure 8.**
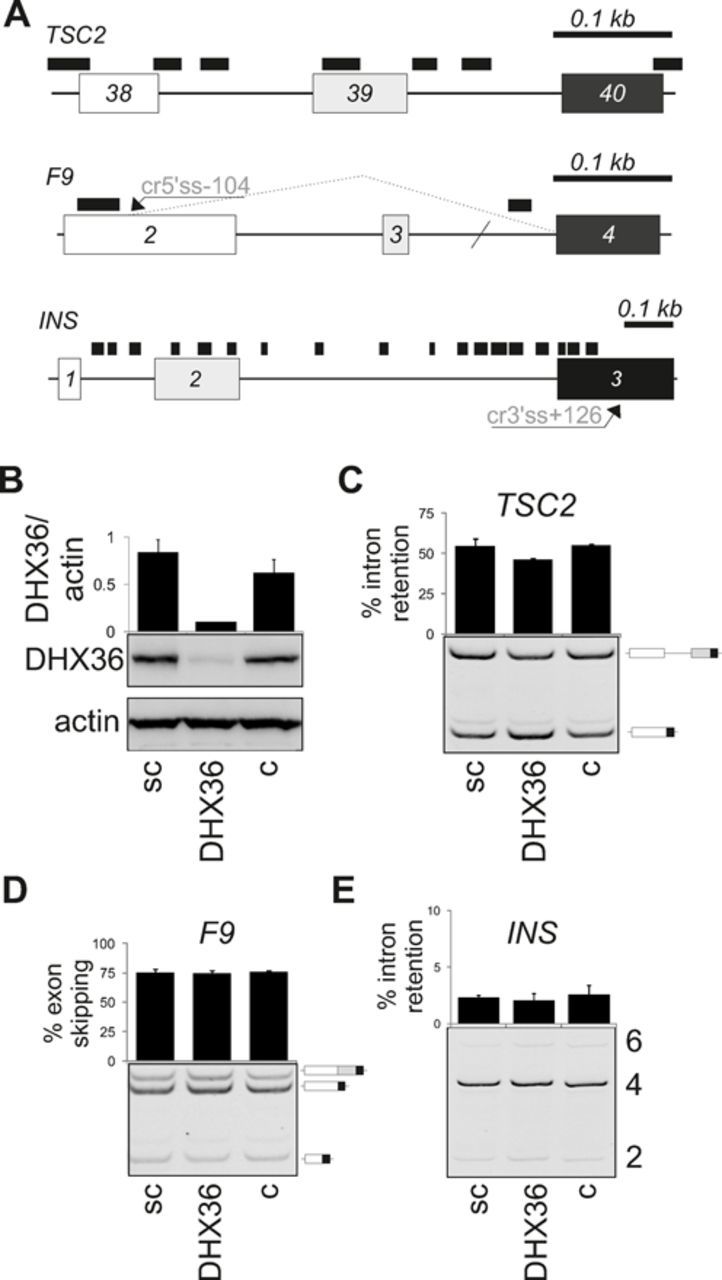
Splicing pattern of quadruplex-rich and -poor minigenes upon DHX36 depletion. (**A**) Schematics of reporter constructs. Predicted quadruplexes are denoted by black rectangles; their densities are shown in Table 1. Exons (boxes) are numbered; forward slash denotes shortening of *F9* intron 3 (24). The *F9* and *TSC2* minigenes contain branch point substitutions c.253–25C and c.5069–18C, respectively, that impair splicing (24). Cr5' ss-104; cryptic 5' ss 104 upstream of authentic 5' ss of intron 2. (**B**) Immunoblot with antibodies against DHX36. sc, scrambled siRNA; c, untreated cells. Error bars are SDs of two transfection experiments. (**C–E**) Intron retention and exon skipping of the indicated reporters. The final concentration of DHX36 siRNA was 50 nM. RNA products are shown schematically to the right. Error bars are SDs of two transfection experiments.

RNA G-quadruplexes bind helicase DHX36, which is capable of converting quadruplex RNA to a stable duplex and is a major source of quadruplex-resolving activity in HeLa cells (26,47). DHX36 was crosslinked to an intronic splicing enhancer in the *ATM* pre-mRNA (48) and could unwind the quadruplex structure within the 5' region of *TERC* (26). To test if DHX36 depletion can influence *INS* splicing, we transiently transfected G-quadruplex-poor and -rich reporters (Figure 8A, Table 1) into depleted cells. Control constructs were chosen to give approximately equal representation of spliced products, which was achieved by weakening the branch site (24), thus providing a sensitive *ex vivo* splicing assay. However, despite efficient DHX36 depletion (Figure 8B), we did not see statistically significant alterations of *INS* intron 1 retention in either short or long constructs, nor did we observe major changes in G-poor and G-rich controls (Figure 8C–E and data not shown). These results are in agreement with a previous lack of significant enrichment of quadruplex sequences among transcripts downregulated in DHX36-depleted cells (49) and with the absence of *ATM* response to the knockdown (48).

**Table 1.** Density of predicted RNA G-quadruplexes in reporter constructs

Reporter	TSC2	F9	INS
G-quadruplexes per nucleotide^a^	0.25	0.05	0.27
G score per nucleotide^a^	0.20	0.04	0.22

^a^The length of non-overlapping quadruplex sequences and their G scores were computed as described (50).

### SSO-induced repression of a population-specific cryptic 5' splice site of *INS* intron 1

In addition to *rs689, INS* intron 1 splicing is influenced by a polymorphic TTGC insertion at *rs3842740* located in the vicinity of the natural 5' ss (21). This insertion is present in a quarter of all African chromosomes but is absent on Caucasian IC haplotypes (20). The insertion activates a downstream cryptic 5' ss (Figure 1A), extending the 5' UTR of the resulting mRNAs by further 26 nucleotides and repressing proinsulin expression (7,21). To test if the new 5' ss can be efficiently inhibited by SSOs, we introduced the same insertion in our IC construct and co-expressed the wild type and mutated reporters with a bridging oligoribonucleotide termed SSO10. Although the cryptic splicing was inhibited, canonical splicing of intron 1 was not completely restored even at high SSO10 concentrations (Supplementary Figure S3 and data not shown), most likely as a result of suboptimal recognition of the authentic 5' ss weakened by the insertion.

To gain initial insights into folding of 5' UTR sequences in the presence and absence of the insertion, we carried out enzymatic structural probing using partial RNA digestion with single- and double-strand specific RNAses (Supplementary Figure S4). The overall cleavage positions and intensities detected for the wild-type RNA were broadly consistent with mfold predictions, in which two major stem-loop regions (SL1 and SL2) were interrupted by several internal bulges. Both the structural probing and mfold predictions suggested that the insertion at *rs3842740* extended the central bulge in SL1 as the number of T1 and S1 cleavages in this region increased in contrast to the remaining portions of SL1 and in SL2 (Supplementary Figure S5). Finally, transcripts were not digested by RNase V1 in regions showing quadruplex formation *in vitro*.

## DISCUSSION

### Antisense intron retention target in a splicing silencer of *INS* intron 1

Here we demonstrate the first use of antisense technology to reduce retention of the entire intron in mature transcripts and to modify the haplotype-dependent *INS* expression using SSOs. Identification of winner SSOs that compensate the adverse impact of the A allele at *rs689* on efficient RNA processing was facilitated by systematic mutagenesis of intron 1 (7), and by our macro- (Figure 1) and micro-walk (Figure 4) strategies. A similar approach was used previously for fine-mapping sequences that influence inclusion of *SMN2* exon 7 in the mRNA (51). Interestingly, the target sequence contains a tandem CAG(G/C) motif, which resembles a 3' ss consensus (Figure 4). Such ‘pseudo-acceptors’ were previously implicated in splice-site repression experimentally (27) and are overrepresented in splicing silencers. For example, the two tetramers are more common among high-confidence 102 intronic splicing silencers (52) and are depleted in 109 enhancers (53) identified by fluorescence activated screen of random 10-mers. The YAG motifs were also more frequent than expected among QUEPASA splicing silencers (54), suggesting that they are important functional components of the retention target. The intervening cytosine tract may also play an important role as the frequency of C_4_ runs among QUEPASA silencers is ∼2 times higher than expected. We also found these motifs in 4% of intronic splicing regulatory elements identified by a systematic screening of sequences inserted at positions –62/–51 relative to a tested 3' ss (55). This study identified an element termed ISS22 (AAATAGAGGCCCCAG) that shared a 3' nonamer (underlined) with the optimal intron retention target. However, unlike an optimal 3' ss recognition sequence of AV3, our pull-down assay coupled with western blotting revealed only a very weak binding if any to U2AF65 (Figure 7C).

### Conformational transition between quadruplex and hairpins in RNA processing control

The antisense target was identified just upstream of a potential G-quadruplex forming RNA whose structure was subsequently confirmed by CD and NMR analysis (Figures 1A and 5). RNA quadruplexes are more stable than their DNA counterparts, have been increasingly implicated in regulation of RNA metabolism (34–35,42–43) and offer unique avenues for drug development (56). The 2-quartet quadruplexes are thermodynamically less stable than their 3- or 4-quartet counterparts and are probably kinetically more labile, yet they still display pronounced stability and may serve as more compliant and dynamic switches between quadruplex and non-quadruplex structures in response to cellular environment (57–59). The winner SSOs may block interactions with *trans*-acting factors, alter higher-order structures, the rate of RNA–protein complex formation or impair conformational transition between the 2-quartet quadruplex and H1/H2 (Figure 5). A similar transition has been recently described for a quadruplex not predicted *ab initio* (45), raising a possibility that additional sequences in the G-rich intron 1 may participate in the equilibria near the antisense target, possibly involving multiple quadruplex motifs and competing stem-loops.

Our binding (Figure 7C) and functional experiments showing the increased intron 1 retention upon hnRNP F/H depletion and the opposite effect upon hnRNP F/H overexpression (7) indicate that these proteins interact with key splicing auxiliary sequences in this intron. In contrast to a previous report concluding that hnRNP F binds directly to the RNA quadruplex (60), hnRNP F has been shown to prevent formation of RNA quadruplexes by binding exclusively single-stranded G-tracts (61). Although preliminary predictions based on primate genomes suggest that the majority of putative quadruplexes are likely to fold into canonical structures (62), future studies will be required to explain how decreased pre-mRNA occupancy by these proteins, presumably promoting quadruplex formation (61), can reduce splicing efficiency.

### RNA quadruplexes in coupled splicing and translational gene expression control

RNA quadruplexes were predicted in ∼8.0% of 5' UTR and were proposed to act as general inhibitors of translation (62,63). *INS* intron 1 is weakly spliced and U2AF35-dependent (7) and a significant fraction of intron 1-containing transcripts is exported from the nucleus (23). This suggests that the RNA G-quadruplex formed by CD1 could influence translation of these mRNAs, which contain a three-amino acid uORF specific for *Homininae* (7). This uORF markedly inhibits proinsulin expression and is located just a few base-pairs downstream, prompting a speculation that the G-quadruplexes can promote translation by sequestering uORFs. As functional 2-quartet quadruplexes are required for activity of internal ribosomal entry sites (57), future studies should also explore the importance of these structures in cap-independent translation of proinsulin transcripts (64).

### Antisense strategies for dependencies in splice-site selection

Apart from canonical mRNA isoform 4, isoforms 2, 3 and 6 (Figure 1B) have been found in expressed sequence tag databases derived from cDNA libraries from insulin-producing tissues (21). This suggests that cryptic splice sites produced by our reporter construct are recognized *in vivo* and that our haplotype-dependent reporter system recapitulates these events accurately in cultured cells no matter whether the cells express or not endogenous insulin. Apart from repressing intron 1-retaining transcripts, optimal SSOs increased utilization of cryptic 3' ss of exon 3 (Figure 2). This undesired effect could be explained by coordination of splicing of adjacent exons and introns, which was observed previously for individual genes and globally (65–69). Also, G-richness downstream transcription start sites have been associated with RNA polymerase II pausing sites (70). Although the two robustly competing 3' ss of intron 2 are likely to respond to non-specific signals that influence RNA folding (Figure 3, Supplementary Table S1), it might be possible to alleviate the observed dependencies and reduce cryptic 3' ss activation using SSO combinations at linked splice sites and examine their synergisms or antagonisms, benefiting from the use of full-gene constructs as opposed to minigenes.

### Multifunctional antisense oligonucleotides to reduce *INS* intron 1 retention

Since the first use of 2' -*O*-methyl-phosphorothioate SSOs (71), this type of chemical modification has been successfully exploited for many *in vitro* and *in vivo* applications (9–10,72). To further fine-tune expression of mRNA isoforms, optimized SSOs can be designed to tether suitable *trans*-acting splicing factors to their target sequences (11,73). An obvious candidate for our system is U2AF35 because intron 1 is weak as a result of relaxation of the 3' ss in higher primates and is further undermined by the A allele at *rs689*, which renders this intron highly U2AF35-dependent (Figure 3) (7). Apart from U2AF35, future bi- or multifunctional antisense strategies can employ binding platforms for splicing factors previously shown to influence *INS* intron 1 and exon 2 splicing, such as Tra2β or SRSF3 (7). Tra2β is likely to bind the SSO6 target which forms a predicted stable hairpin structure with a potent GAA splicing enhancer in a terminal loop (Figure 3B). SRSF3 is required for repression of the cryptic 3' ss of intron 2 (7) and binds pyrimidine-rich sequence with a consensus (A/U)C(A/U)(A/U)C (74). The CAUC motif, which interacts with the RNA-recognition motif of SRSF3 (75), is present just upstream of the cryptic 3' ss.

### Normalizing intron retention levels in human genetic disease

Our results provide an opportunity to use non-genetic means to compensate less efficient splicing and lower *INS* expression from haplotypes predisposing to type 1 diabetes. Common variants such as *rs689* contribute to a great extent to the heritability of complex traits, including autoimmune diseases (76), but their functional and structural consequences are largely unknown. If optimized *INS* SSOs can be safely and efficiently introduced into the developing thymus, this approach may offer a novel preventive approach to promote tolerance to the principal self-antigen in type 1 diabetes. The most obvious candidates for such intervention are mothers who had an affected child homozygous for disease-predisposing alleles at both HLA and *INS* loci. Such genotypes were associated with an extremely high disease risk for siblings (77). Apart from primary prevention of type 1 diabetes, future SSO-based therapeutics might be applicable to patients with significant residual β-cell activity at diagnosis and to those who are eligible to receive β-cell transplants and may benefit from increased intron-mediated enhancement of proinsulin expression from transplanted cells. It is also possible to envisage use of this therapeutic modality for other patients with diabetes through a more dramatic enhancement of intron splicing and proinsulin expression by targeting multiple splicing regulatory motifs with multifunctional SSOs. Future studies should therefore examine utility of our SSOs in thymic epithelial cells and β-cells that may provide a more natural system for testing their impact on both exo- and endogenous proinsulin expression. Finally, similar antisense strategies may help reduce pervasive intron retention in cancer cells resulting from somatic mutations of splicing factor genes, as illustrated by specific substitutions in the zinc finger domain of U2AF35 in myeloproliferative diseases (78).

## SUPPLEMENTARY MATERIAL

Supplementary Data are available Online.

SUPPLEMENTARY DATA
