# Supplementary material for: Optimal antisense target reducing INS intron 1 retention is adjacent to a parallel G quadruplex
Source: Nucleic Acids Res. 2014 Jun 17;42(12):8161–73. doi: 10.1093/nar/gku507 (PMC4081105; doi:10.1093/nar/gku507)
Supplement: SUPPLEMENTARY DATA [file supp_42_12_8161__index.html]

Optimal antisense target reducing INS intron 1 retention is adjacent to a parallel G quadruplex — Optimal antisense target reducing INS intron 1 retention is adjacent to a parallel G quadruplex — Optimal antisense target reducing INS intron 1 retention is adjacent to a parallel G quadruplex — SUPPLEMENTARY DATA 

# Optimal antisense target reducing *INS* intron 1 retention is adjacent to a parallel G quadruplex

## SUPPLEMENTARY DATA

**Files in this Data Supplement:**

- SUPPLEMENTARY DATA
