# Supplementary material for: Optimal antisense target reducing INS intron 1 retention is adjacent to a parallel G quadruplex
Source: Nucleic Acids Res. 2014 Jun 17;42(12):8161–73. doi: 10.1093/nar/gku507 (PMC4081105; doi:10.1093/nar/gku507)
Supplement: SUPPLEMENTARY DATA [file supp_gku507_nar-00832-y-2014-File003.pdf]

SUPPLEMENTARY DATA

Figures S1-S5

Tables S1 and S2

Optimal antisense target reducing *INS* intron 1 retention is adjacent to a parallel G quadruplex

Jana Kralovicova[1], Ana Lages[1], Alpa Patel[2], Ashish Dhir[3],

Emanuele Buratti[3], Mark Searle[2], Igor Vorechovsky[1]

[1]University of Southampton
Faculty of Medicine
Southampton SO16 6YD
United Kingdom

[2]University of Nottingham
School of Chemistry
Centre for Biomolecular Sciences
Nottingham NG7 2RD
United Kingdom

[3]ICGEB,
Padriciano 99,
34149 Trieste,
Italy

FIGURE S1 SSO21-induced decrease of *INS* intron 1 retention in HepG2 cells

Legend: Final SSO concentrations were the same as in Fig. 2A. SCm, scrambled controls, SSO-, no-SSO control.

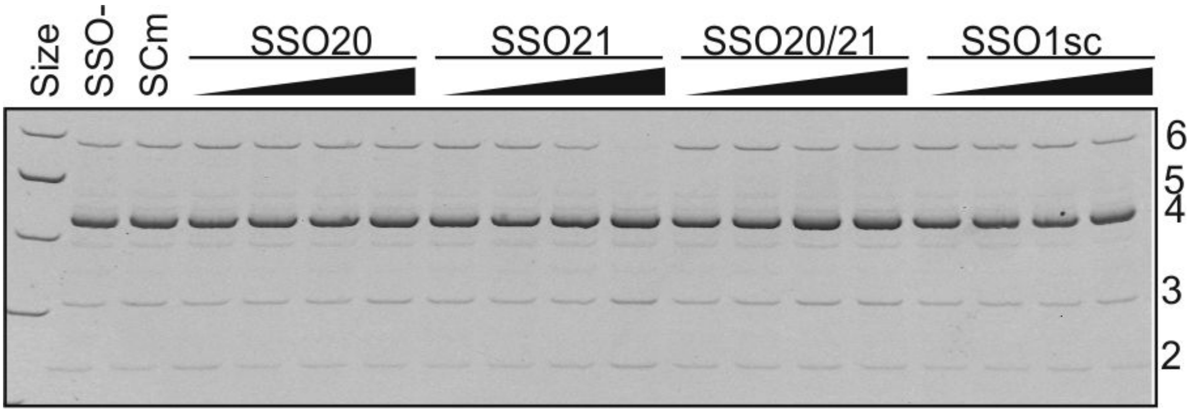

FIGURE S2 Prediction of putative enhancer binding sites of SR proteins in the region surrounding the intron 1 antisense target

Legend: The prediction was carried out by ESEFinder using default score matrices (1). SRSF1/SRSF1(IgM-BRCA1) sites are shown in red/purple, SRSF2 in blue, SRSF5 in green and SRSF6 in yellow. The SSO21 target sequence is shown by a box.

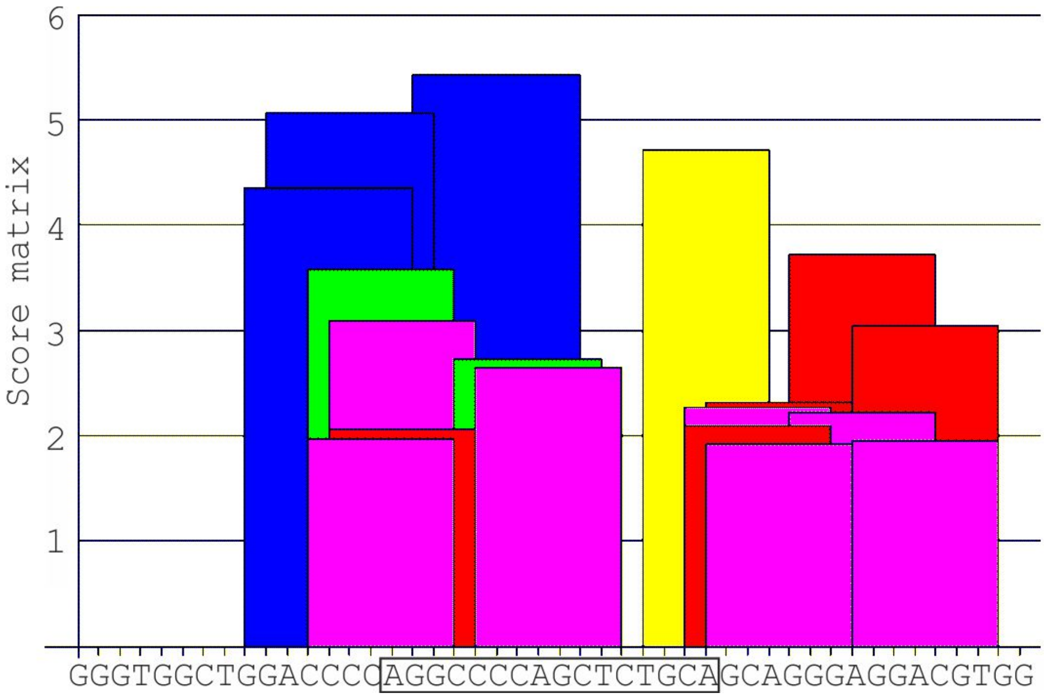

FIGURE S3   A partial restoration of authentic 5’ splice site of *INS* intron 1

Legend: RNA products are shown to the right, the reporter at the bottom. SSOs are shown at the top, the final concentration of SSO10 in transfections wells were 10, 30 and 90 nM.

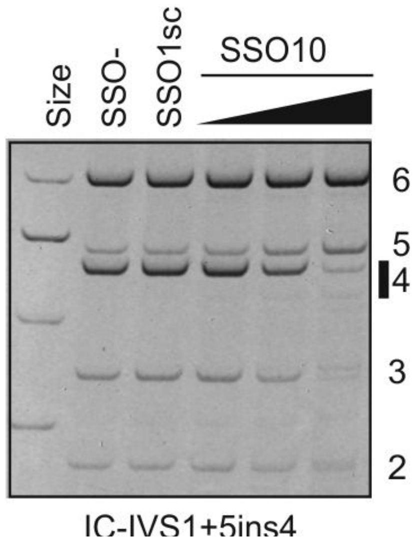

FIGURE S4   Enzymatic probing of *INS* 5'UTR

Legend: *In vitro* transcribed RNA enzymatically digested with S1 nuclease, T1 and V1 RNases. No enzyme was added to the RNA in a control reaction mixture (lane C).The cleaved fragments were detected by an RT reaction with an isotopically labelled oligonucleotides. The RT products were separated on a denaturing 6% polyacrylamide gel. A sequencing reaction performed with the same RT primer was run in parallel (lanes g, a, t and c). Squares, circles and triangles indicate S1, T1 and V1 cleavage sites. Black and white symbols indicate high and low cleavage intensity, respectively. The positions of the splice sites are indicated by arrows and their intrinsic strength by Shapiro-Senapathy scores (2). Secondary structure predictions were carried out using mfold (3).

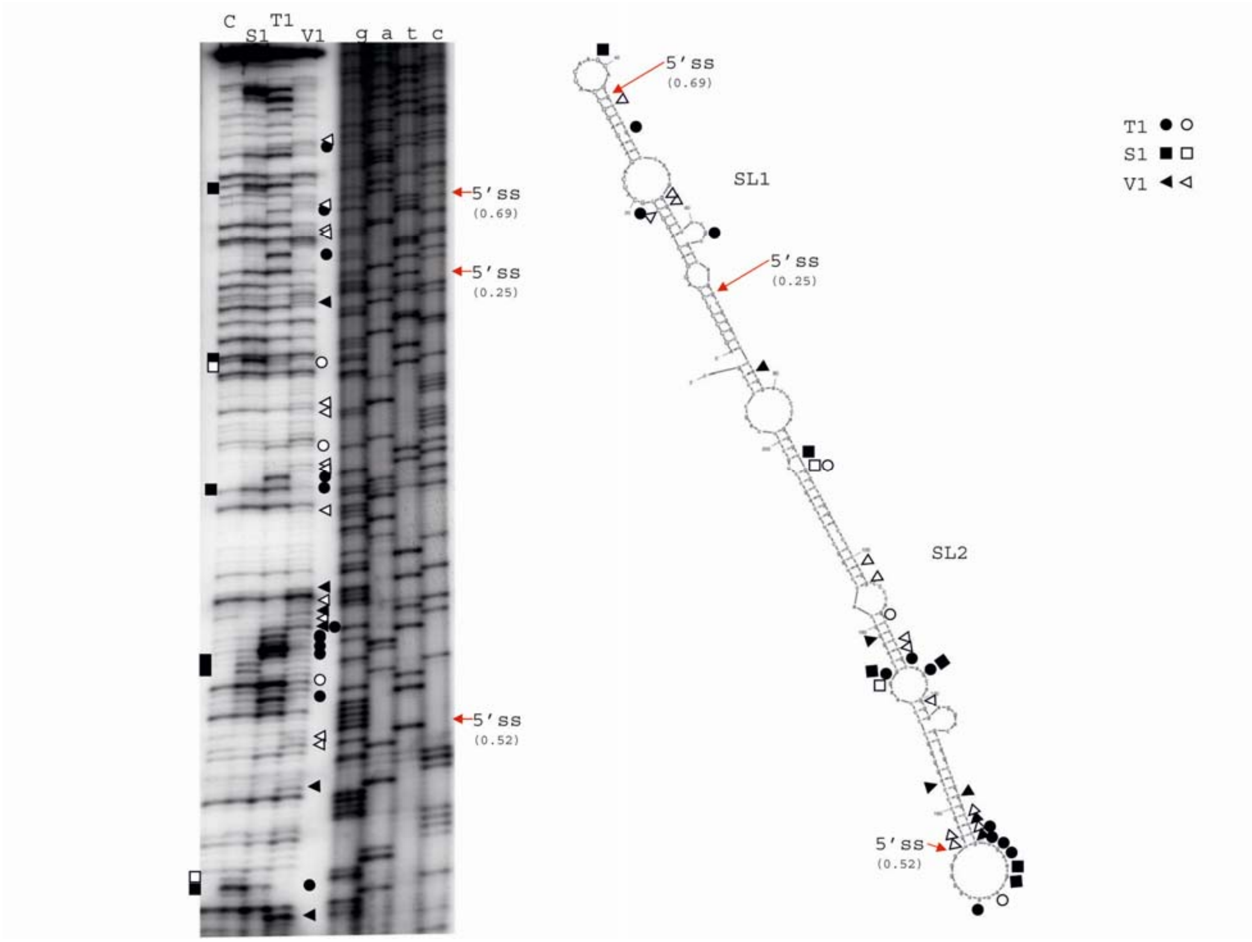

FIGURE S5 Structural probing of ins/del RNAs at *rs3842740*

For legend, see Figure S4.

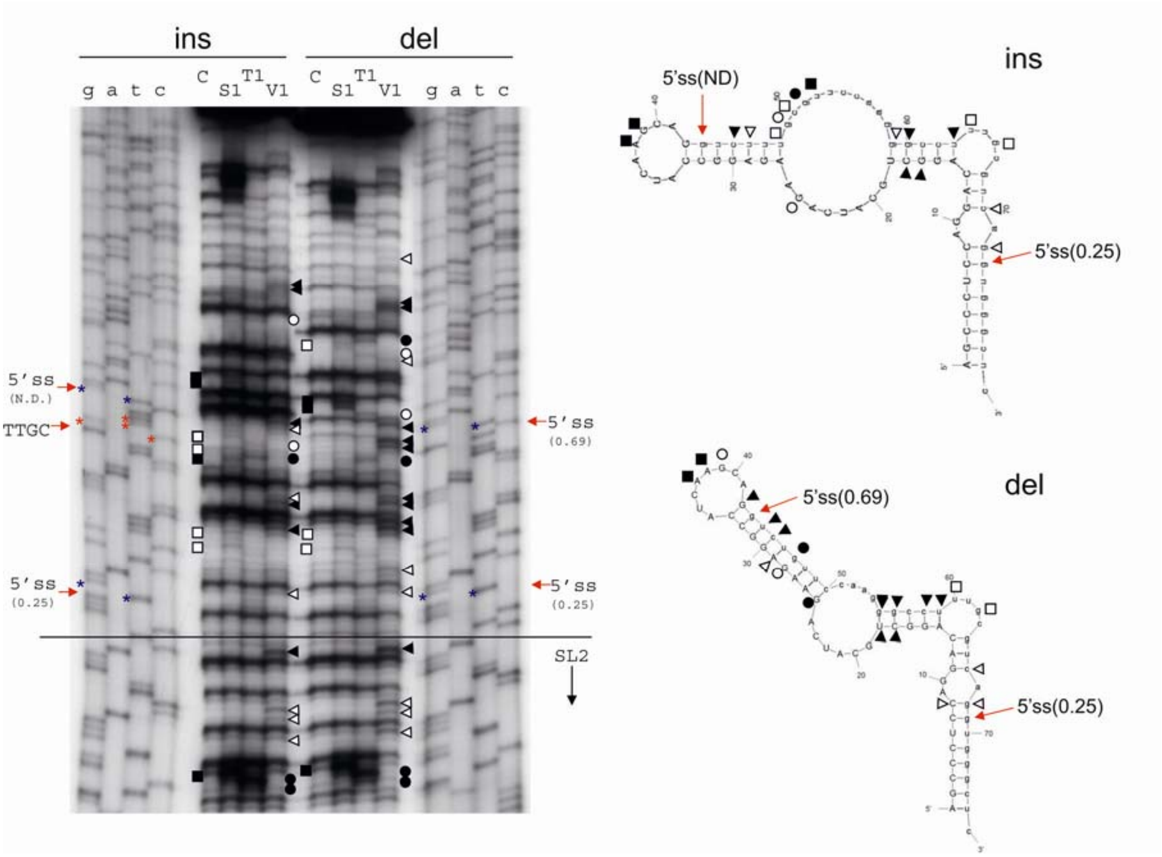

TABLE S1 Splice-switching oligonucleotides targeting proinsulin pre-mRNAs

SSO	Location[1]	Sequence (5'-3')	Effects on the relative abundance of *INS* mRNA isoforms
1	Intron 1 (del5, del6)	AGCUGGGGCCUGGGGU	Activation of the cryptic 3'ss of intron 2
2	Intron 1 (del5, del6)	UGCAGAGCUGGGGCCUGGGGU	Activation of the cryptic 3'ss of intron 2
3	Intron 1 (del8, del9)	CAUGCUUCACGAGCCCAGCC	Increased exon 2 skipping
4	Exon 2 (cryptic 3'ss +81, del8, del9)	AAGGCUGCGGCUGGGUC	Increased exon 2 skipping
5	Exon 3	UGGUAGAGGGAGCAGAUGCUG	Decreased efficiency of intron 2 splicing; Activation of the cryptic 3'ss of intron 2
6	Exon 3	UGGUACAGCAUUGUUCCACA	Activation of the cryptic 3'ss of intron 2 at high concentration
8	Exon 2 (del13-15)	CGCACACUAGGUAGAGAGC	Increased exon 2 skipping
9	Exon 1	GAUGCAGCCUGUCCUGGAG	None
10	Intron 1 (del1, del2, cryptic 5' splice site +30)	GAGCCCACCUGACGCAAAGGC	Partial restoration of authentic 5' splice site
16	Exon 1	UGGAGGGCUGAGGGCUGCU	None
17	Exon 1	AUGGCCUCUUCUGAUGCA	None
18	Intron 1 (del9, del10)	UCACCCCCACAUGCUUC	Increased exon 2 skipping
19	Intron 1 (del9)	ACAUGCUUCACGAG	Increased exon 2 skipping
20	Intron 1 (del5)	CUGGGGCCUGGGGU	Minor reduction of intron 1 retention; activation of the cryptic 3'ss of intron 2
21	Intron 1 (del5, del6)	UGCAGAGCUGGGGCCU	Reduction of intron 1 retention; activation of the cryptic 3'ss of intron 2
1sc	Scrambled control	AGGUGCUCGCGGGUGG	None
2sc	Scrambled control	GGGUGGAAGCGUCCGGUCGUG	Stimulation of the cryptic 3'ss of intron 2
3sc	Scrambled control	ACACACUGUGCCUCGCCAGC	None
6sc	Scrambled control	GACUCACUUGCCGUAGUUAA	Stimulation of the cryptic 3'ss of intron 2
8sc	Scrambled control	CACGCUCAGUAGAGAAGGC	None

[1], sequence of deleted segments (del) is shown in Fig. 2 and Supplemental Fig. 13 of our previous study (4).

TABLE S2 *INS* intron 1 mutatons altering predicted RNA G quadruplexes, stem loops and two cytosines runs in plasmid constructs

Mutation	Input sequence for computation predictions[1]	Predicted RNA quadruplex	H2	H1	C runs	The most stable RNA structure	Free energy (kcal/mmol)
Wildtype sequence	GGAUUCCAGGGUGGCUGGACCCCAGGCCCC	+	+	+	+	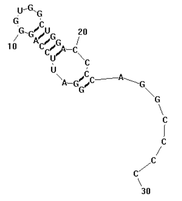	-9.8
Del5	GGAUUCCAGGGUGGCUGG - - - - - - - - - - - -	+	+	-	-	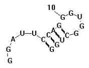	-2.7
M1	GGAUUCCAGGGUGGCUGCACGCCAGGCCCC	+	-	-	-	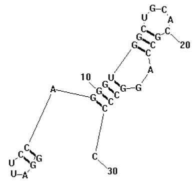	-9.3
1	GGAUUCCAGGGUGGCUGGACCCGAGGCGCC	+	+	+	-	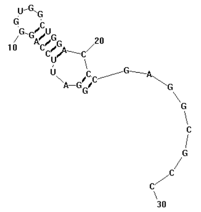	-8.4
2	GGAUUCCAGGAUGGUAGGACCCGAGGCGCC	+	-	-	-	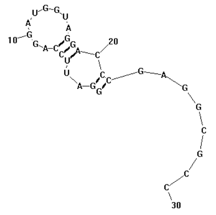	-2.8
3	GGAUUCCAGGAUGGUAGGACCCCAGGCCCC	+	-	-	+	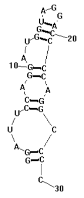	-4.3
4	GGAUUCCAGGGUUGCUGGACCCCAGUCCCC	-	+	+	+	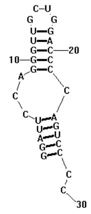	-11.1
5	GGAUUCCAGGGUUGCUGGACCCGAGUCGCC	-	+	+	-	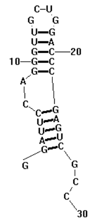	-11.3

6	GGAUUCCAGGAUUGUAGGACCCCAGUCCCC	-	-	-	+	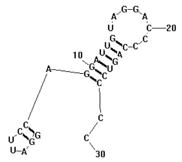	-6.4
8	GGAUUCCAGGGUGGCUGGACUUCAGGCCCC	+	+	-	-	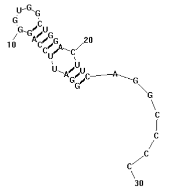	-9.0
9	GGAUUCCAGGGUGGCUGGACCCCAGGCUUC	+	+	+	-	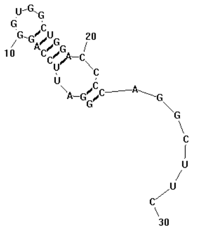	-9.8
10	GGAUUCCAGGGUGGCUGGACUUCAGGCUUC	+	+	-	-	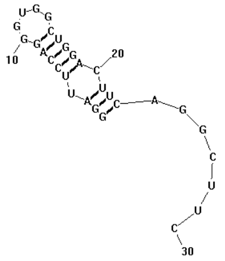	-9.0

[1]sequences that form H1 (strikethrough) and H2 (underlined) stems are highlighted. Guanines contributing to predicted quadruplex formation are in yellow; mutations are in red. RNA secondary structures/free energy were predicted by RNAStructure (v.5.2) (5).

References to supplementary data

1 Cartegni, L., Wang, J., Zhu, Z., Zhang, M.Q. and Krainer, A.R. (2003) ESEfinder: a web resource to identify exonic splicing enhancers. Nucleic Acids Res., 31, 3568-3571.

2 Shapiro, M.B. and Senapathy, P. (1987) RNA splice junctions of different classes of eukaryotes: sequence statistics and functional implications in gene expression. Nucleic Acids Res., 15, 7155-7174.

3 Zuker, M. (2003) Mfold web server for nucleic acid folding and hybridization prediction. Nucleic Acids Res., 31, 3406-3415.

4 Kralovicova, J. and Vorechovsky, I. (2010) Allele-dependent recognition of the 3' splice site of INS intron 1. Hum. Genet., 128, 383-400.

5 Reuter, J.S. and Mathews, D.H. (2010) RNAstructure: software for RNA secondary structure prediction and analysis. BMC Bioinformatics, 11, 129.
